# Correction: Fungal and host protein persulfidation are functionally correlated and modulate both virulence and antifungal response

**DOI:** 10.1371/journal.pbio.3001381

**Published:** 2021-08-13

**Authors:** The PLOS Biology Staff

The incorrect file for S2 Table was uploaded. Please see the correct S2 Table. The publishers apologise for the error.

## Supporting information

S2 Table List of proteins of the persulfidated enriched fraction identified by mass spectrometry using the A. fumigatus Z5 annotation in the Swiss-Prot Trembl database. (XLSX)
