# Supplementary figures and images for: Fungal and host protein persulfidation are functionally correlated and modulate both virulence and antifungal response
Source: PLoS Biol. 2021 Jun 1;19(6):e3001247. doi: 10.1371/journal.pbio.3001247 (PMC8168846; doi:10.1371/journal.pbio.3001247)

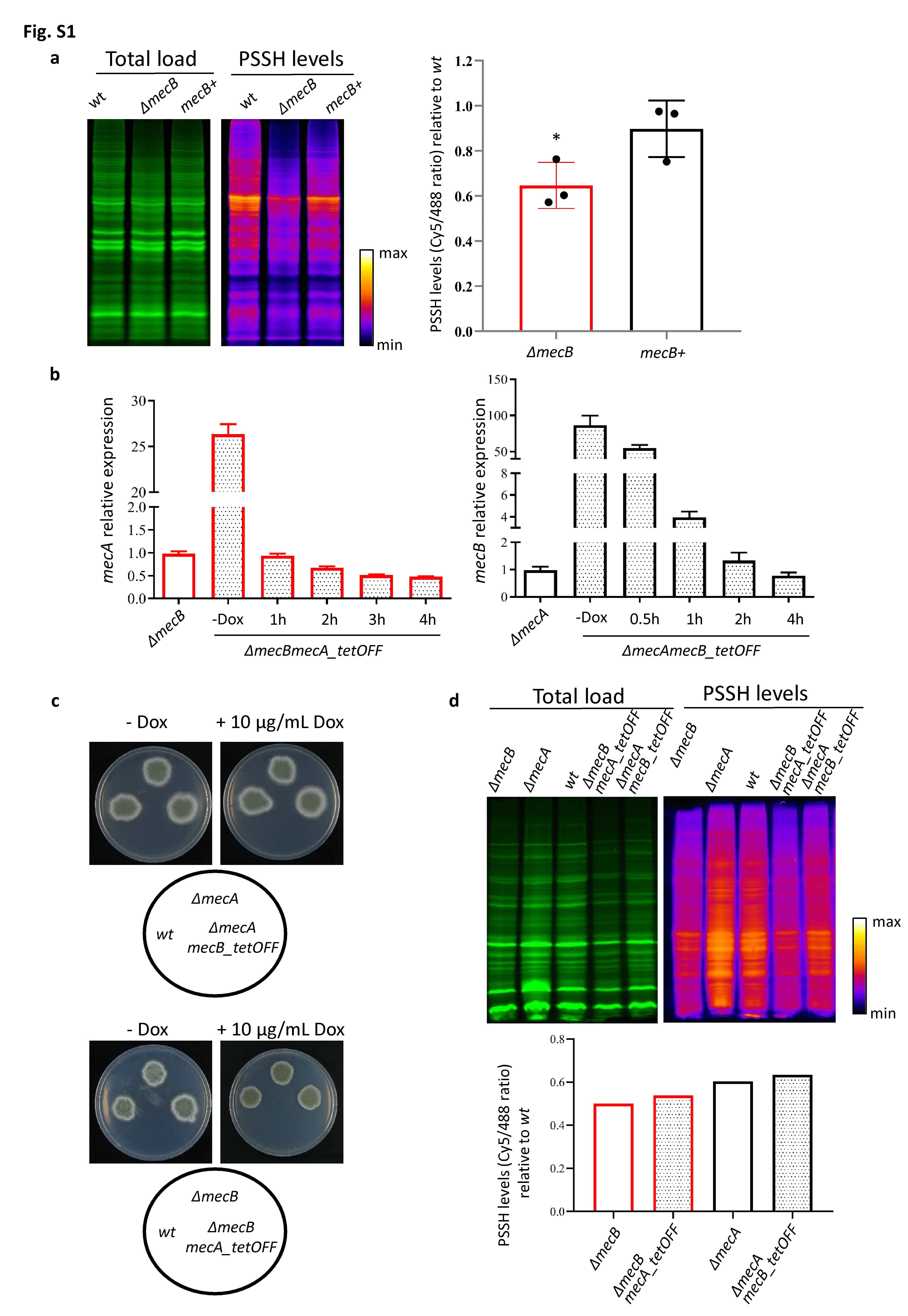

Supplement: S1 Fig — (a) Reintroduction of mecB gene reconstituted the persulfidation levels, showing that the effect is specific to gene deletion (n = 3). All data in are depicted as mean ± SD and were analyzed using 1-way ANOVA with Dunnett multiple comparisons. (b) RT-PCRs to measure mecA (left panel) and mecB (right panel) gene expression relative to transcription in the background single mutant strain (n = 3). For both genes, the basal gene expression under the TetOFF promoter was much higher than native expression. Addition of Dox strongly reduced transcription, but not to expression levels significantly lower than the native expression of the gene. (c) The TetOFF strains grew normally in the presence of Dox. (d) Persulfidation levels of the TetOFF strains was similar to that of their single mutant background mutants (n = 1). All numerical values that underlie the data displayed in this figure can be found in S5 Data. Dox, doxycycline; RT-PCR, reverse transcription polymerase chain reaction; SD, standard deviation. (TIF) [file pbio.3001247.s001.tif]

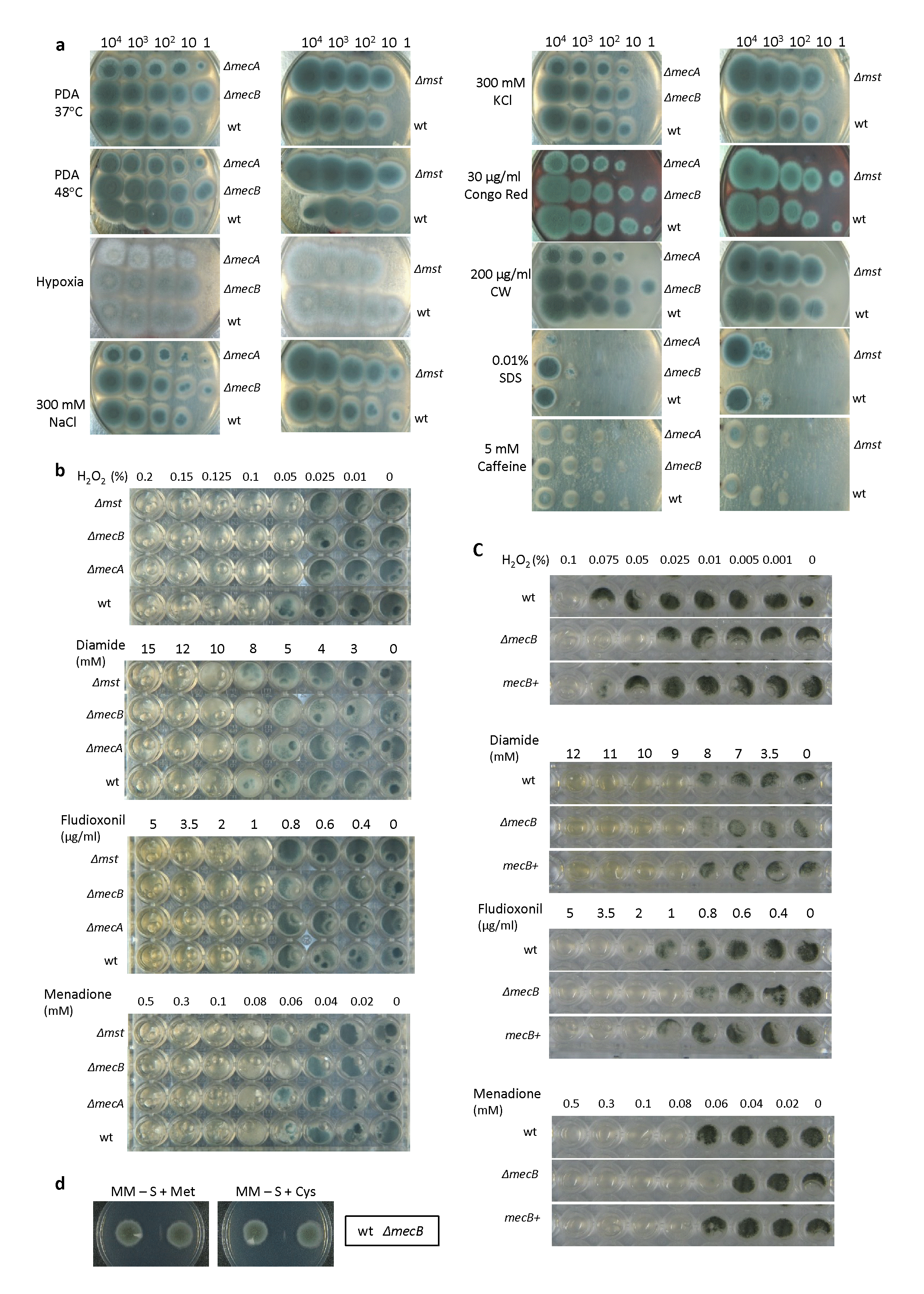

Supplement: S2 Fig — (a) A. fumigatus mutants were not sensitive to high temperature (48°C), hypoxia, or osmotic stress (300 mM NaCl and KOH). The ΔmecA mutant was slightly more sensitive to the cell wall stressor SDS (0.01%), but not Congo Red (30 μg/ml), CalcofluorWhite (200 μg/ml), and Caffeine (5 mM). The phenotype was repeated in 2 independent experiments. (b) All mutants were more sensitive to H202 and Fludioxonil and the ΔmecB mutant was also more sensitive to Menadione and slightly to Diamide. (c) Reconstitution of mecB in its natural locus (mecB+) restored wt levels of resistance to all oxidative stressors. The sensitivity to oxidative stressors has been assayed in 3 independent experiments. (d) The ΔmecB mutant grew as the wt on methionine or cysteine (5 mM) as the sole sulfur source, indicating that absence of the CTH is not impactful on sulfur metabolism. The plates were repeated in 2 independent experiments. CTH, cystathionine γ-lyase; wt, wild-type. (TIF) [file pbio.3001247.s002.tif]

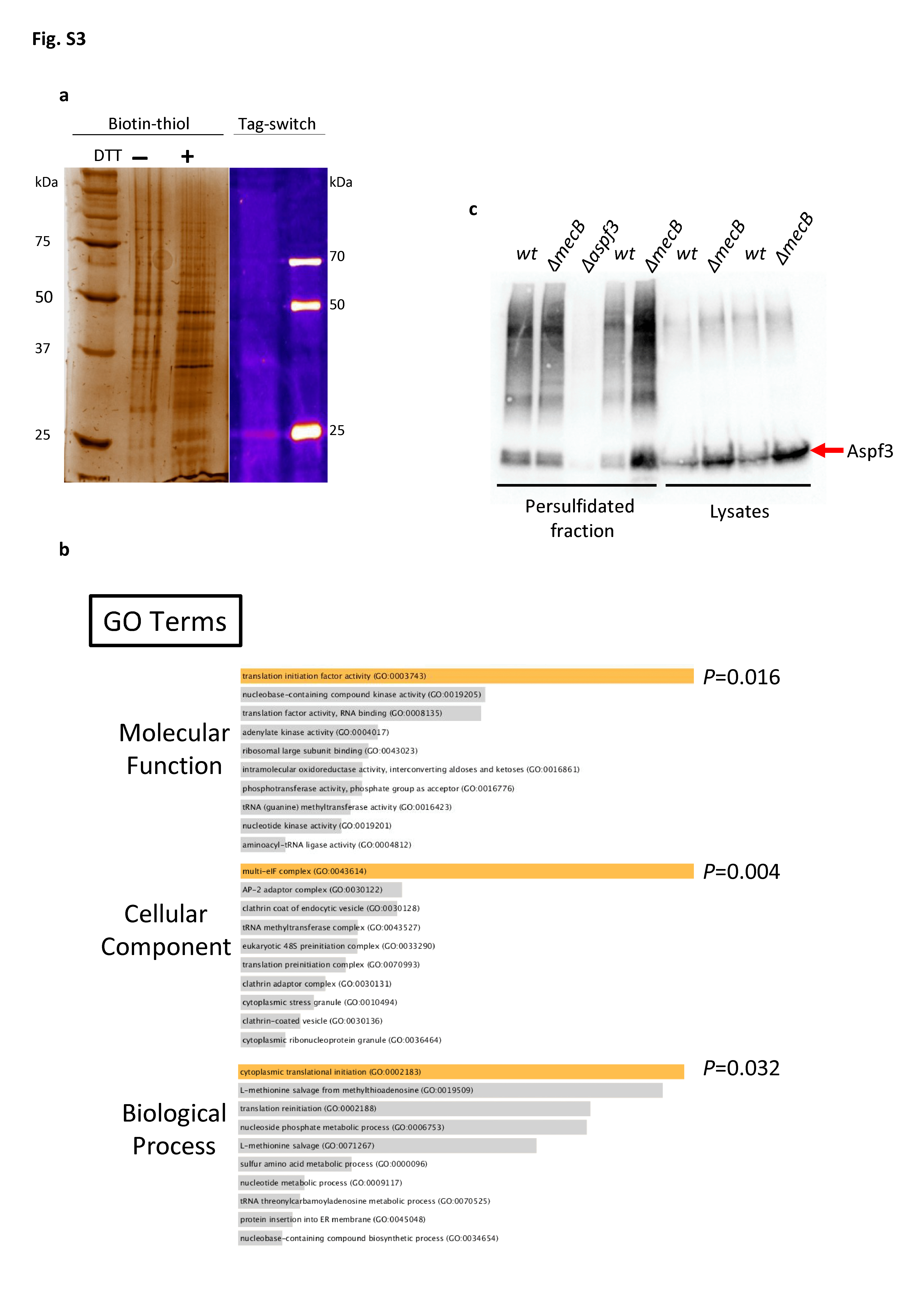

Supplement: S3 Fig — (a) Persulfidated proteins were detected by 2 different methods, the biotin-thiol assay (persulfidated proteins are eluted in the presence of DTT) and the Tag-switch. (b) Pathway enrichment analysis of persulfidated proteins using the YeastEnrichr platform showed a significant enrichment of GO terms. (c) Representative western blot of full lysates and persulfidated enriched fraction of wt and ΔmecB protein extracts using an Aspf3 antiserum. All numerical values that underlie the data displayed in this figure can be found in S6 Data. GO, Gene Ontology; wt, wild-type. (TIF) [file pbio.3001247.s003.tif]

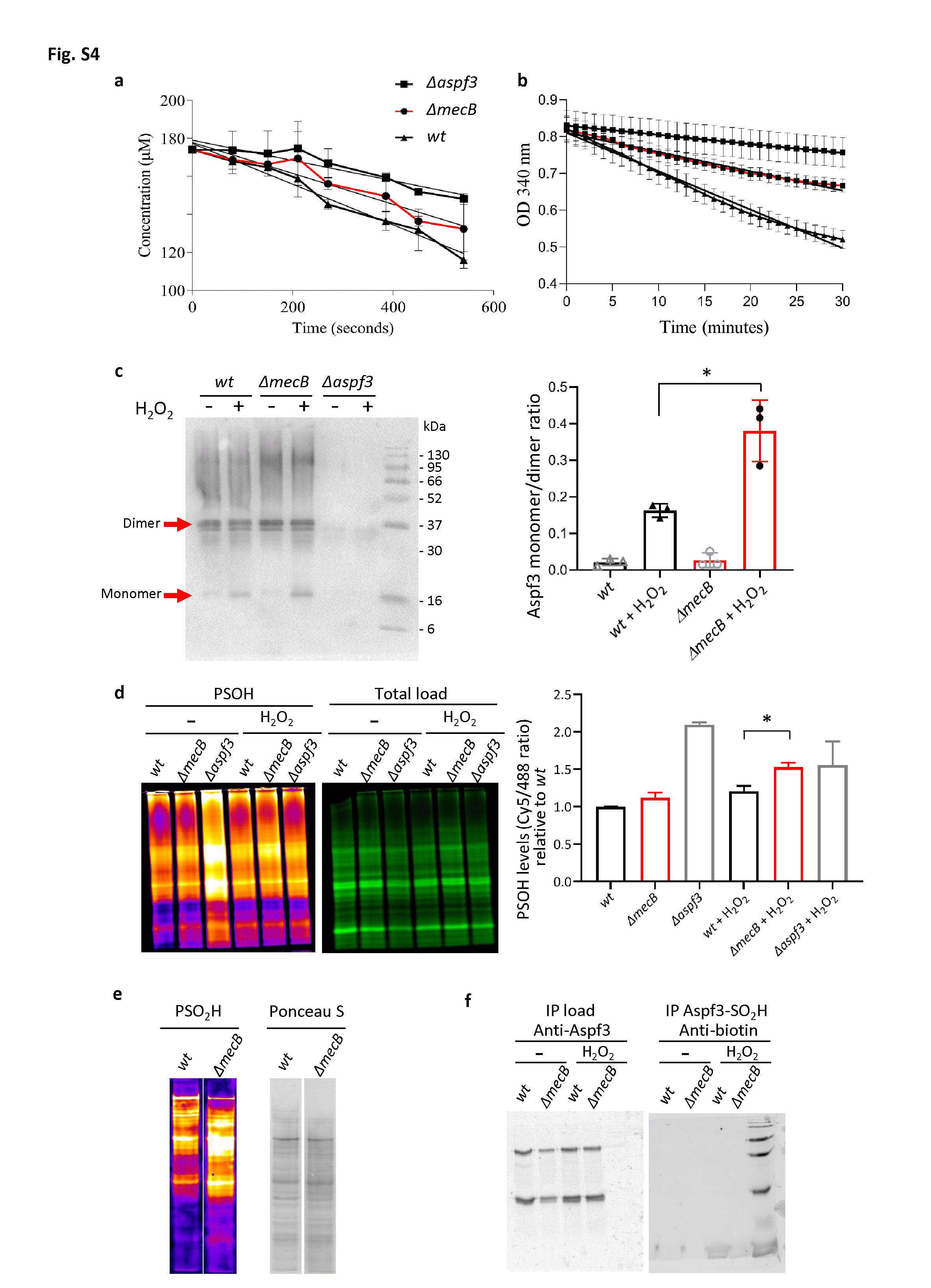

Supplement: S4 Fig — (a) Degradation rate of tert-Butyl hydroperoxide reflects H2O2 detoxifying activity. The Δaspf3 mutant, a control of reduced peroxiredoxin activity, showed a strong reduction in degradation rate. The ΔmecB mutant also had a lower degradation rate. The curve shows one representative experiment with 3 technical replicates. (b) The ratio of NADPH oxidation, and consequent decrease in absorbance at 340 nm, reflects peroxiredoxin activity in the thioredoxin-dependent assay. Both ΔmecB and Δaspf3 had a strong reduction in enzymatic activity. The curve shows 3 biological replicates. (c) Representative nonreducing western blot, used to differentiate between Asp3 monomers and dimers. Two bands of dimer Aspf3 can be observed (not in the Δaspf3 control), likely because Aspf3 can form homodimers and heterodimers with other peroxiredoxins. Quantification of the ratio of monomers (= oxidized) and dimers (= reduced) showed that the oxidation status of Aspf3 is higher in ΔmecB (n = 3, P = 0.04, unpaired t test with Welch correction). (d) Representative gel and quantification of PSOH. In the presence of H2O2, the ΔmecB mutant showed significantly higher levels of oxidation than the wild type (n = 2, wild type+H2O2 vs. ΔmecB+H2O2 P = 0.04, unpaired 2-tailed t test). (e) Total protein fraction was labeled with Biotinylated-Dia-Alk probe (binds PSO2H) and blotted with Streptavidin DyLight 633. In the presence of H2O2, the level of hyperoxidation (sulfinylation) was clearly higher in the ΔmecB mutant than in wild type (n = 1). (f) Total protein fraction was labeled with Biotinylated-Dia-Alk probe and IP with the Anti-Aspf3 antiserum. The IP fraction was blotted with Anti-Aspf3 as IP and load control and with Anti-biotin antibody to detect hyperoxidized (sulfinylated) Aspf3. No hyperoxidized Aspf3 could be detected (representative blot of n = 2). All numerical values that underlie the data displayed in this figure can be found in S7 Data. IP, immunoprecipitated; PSOH, protein sulfenylati [file pbio.3001247.s004.tif]

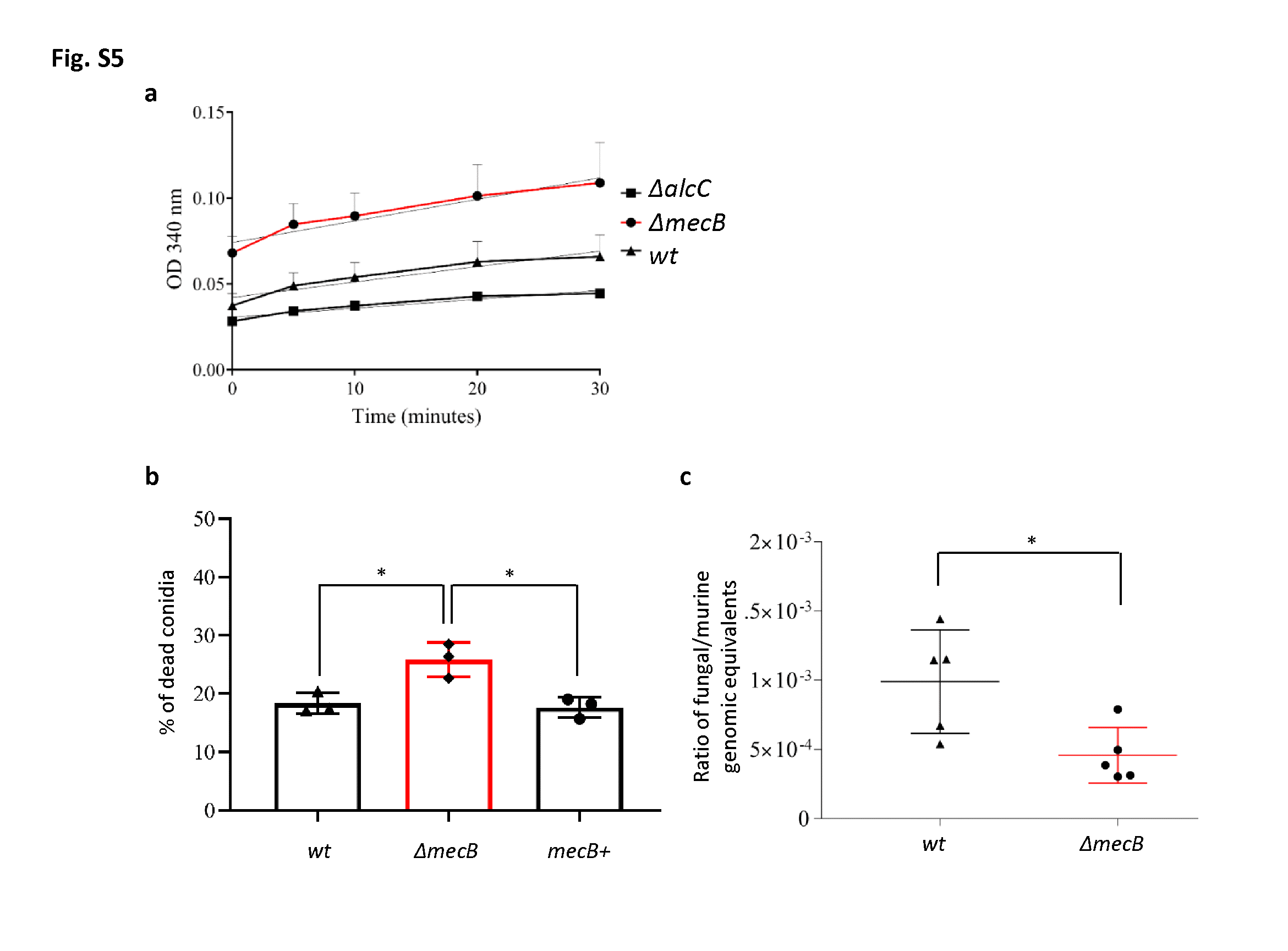

Supplement: S5 Fig — (a) The ratio of NADP+ reduction to NADPH reflects alcohol dehydrogenase enzymatic activity and is measured by NADPH absorbance at 340 nm. The ΔalcC mutant showed a strong reduction in NADPH production. The ΔmecB mutant had a slightly increased speed in NADPH production. The curve shows one representative experiment with 3 technical replicates. (b) The mecB+ reconstituted strain was killed by Raw.264.7 at the same levels as the wild type strain (P = 0.92, 1-way ANOVA with Tukey multiple comparisons) (n = 3 with 3 technical replicates). (c) Lungs of leukopenic mice infected with the ΔmecB mutant showed a decreased fungal burden compared to wild type infected (P = 0.03), unpaired 2-tailed t test with Welch correction) (n = 5, 3 technical replicates per qPCR). Data are depicted as mean ± SD. All numerical values that underlie the data displayed in this figure can be found in S8 Data. SD, standard deviation. (TIF) [file pbio.3001247.s005.tif]

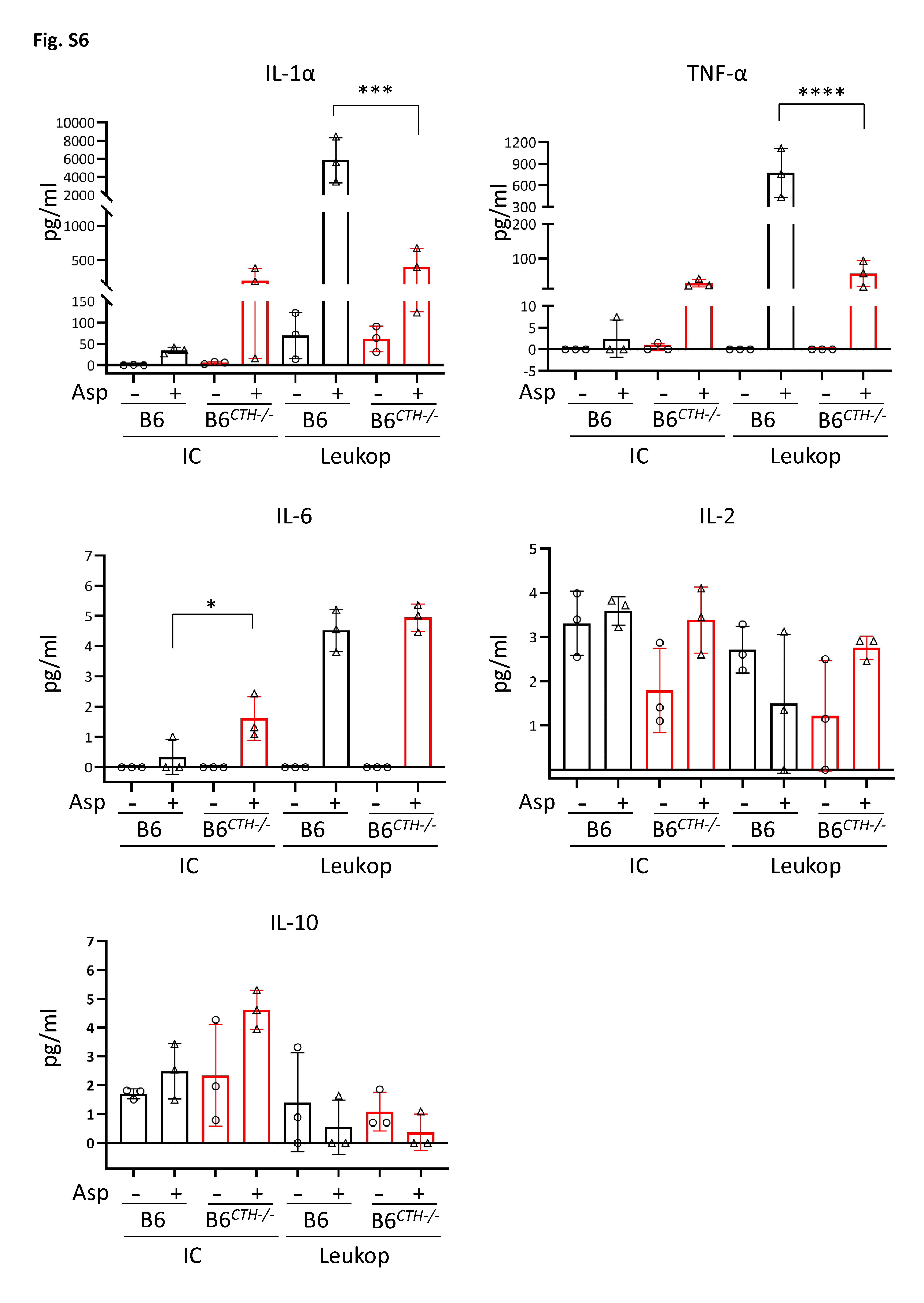

Supplement: S6 Fig — In leukopenic mice, the pro-inflammatory cytokines IL-1α (P = 0.0005) and TNFα (P = 0.0006) were significantly elevated in C57BL/6CTH−/− compared with wild-type mice (n = 3 mice, with 2 technical replicates). All data are displayed as mean ± SD and analyzed using 1-way ANOVA with Tukey multiple comparisons. All numerical values that underlie the data displayed in this figure can be found in S9 Data. CTH, cystathionine γ-lyase; IC, immunocompetent; IL, interleukin; SD, standard deviation; wt, wild-type. (TIF) [file pbio.3001247.s006.tif]

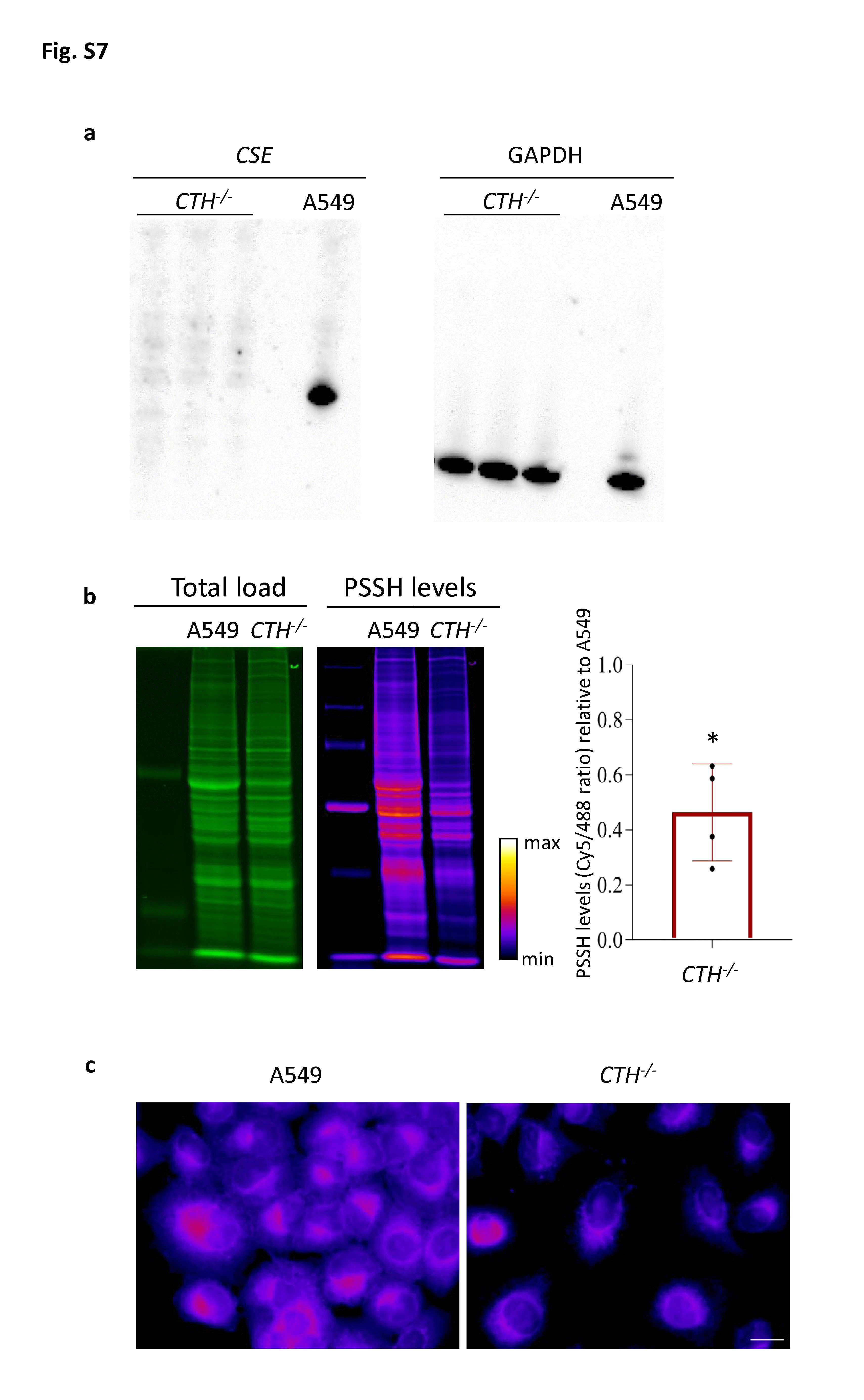

Supplement: S7 Fig — (a) Representative western blot of the CTH protein in the A549 and its derivative CTH−/− epithelial cell lines. GAPDH was used as loading control. (b) Representative image of in-gel detection of persulfidation levels in A549 and CTH−/− cell lines. NBF-Cl labels persulfides, thiols, sulfenic acids, and amino groups; reaction with amino groups produces the green signal; therefore, it reflects the whole protein context and is used to normalize the persulfidation levels. The red signal is produced by the dimedone-Cy5 labeled probe, which selectively switches NBF-Cl in persulfide groups [20]. Quantification of persulfidation levels, measured as the level of red signal normalized to the green signal, showed a significant decrease (P = 0.009) in persulfidation level of CTH−/− relative to A549 (n = 4). Data were analyzed using a 1-sample t test. (c) Representative images (scale bar = 15 μm) of persulfidation levels detected by microscopy. All numerical values that underlie the data displayed in this figure can be found in S10 Data. CTH, cystathionine γ-lyase; GAPDH, glyceraldehyde 3-phosphate dehydrogenase; NBF-Cl, 4-chloro-7-nitrobenzofurazan. (TIF) [file pbio.3001247.s007.tif]

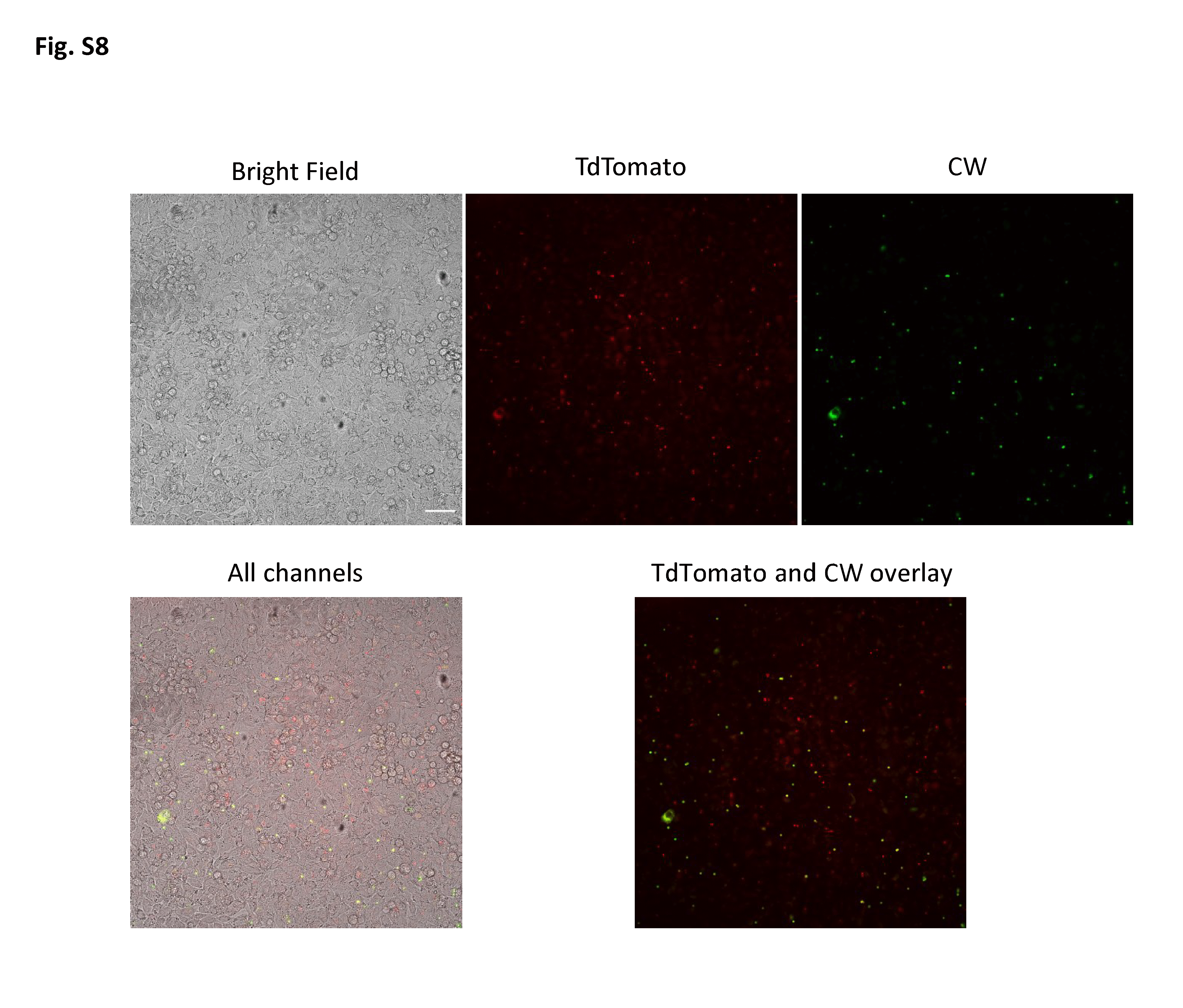

Supplement: S8 Fig — Red color displays the TdTomato signal, which constitutes the whole conidia population. Green color displays the Calcofluor White signal, which constitutes the extracellular (i.e., non-phagocytosed) conidia population. Scale bar = 30 μm. (TIF) [file pbio.3001247.s008.tif]

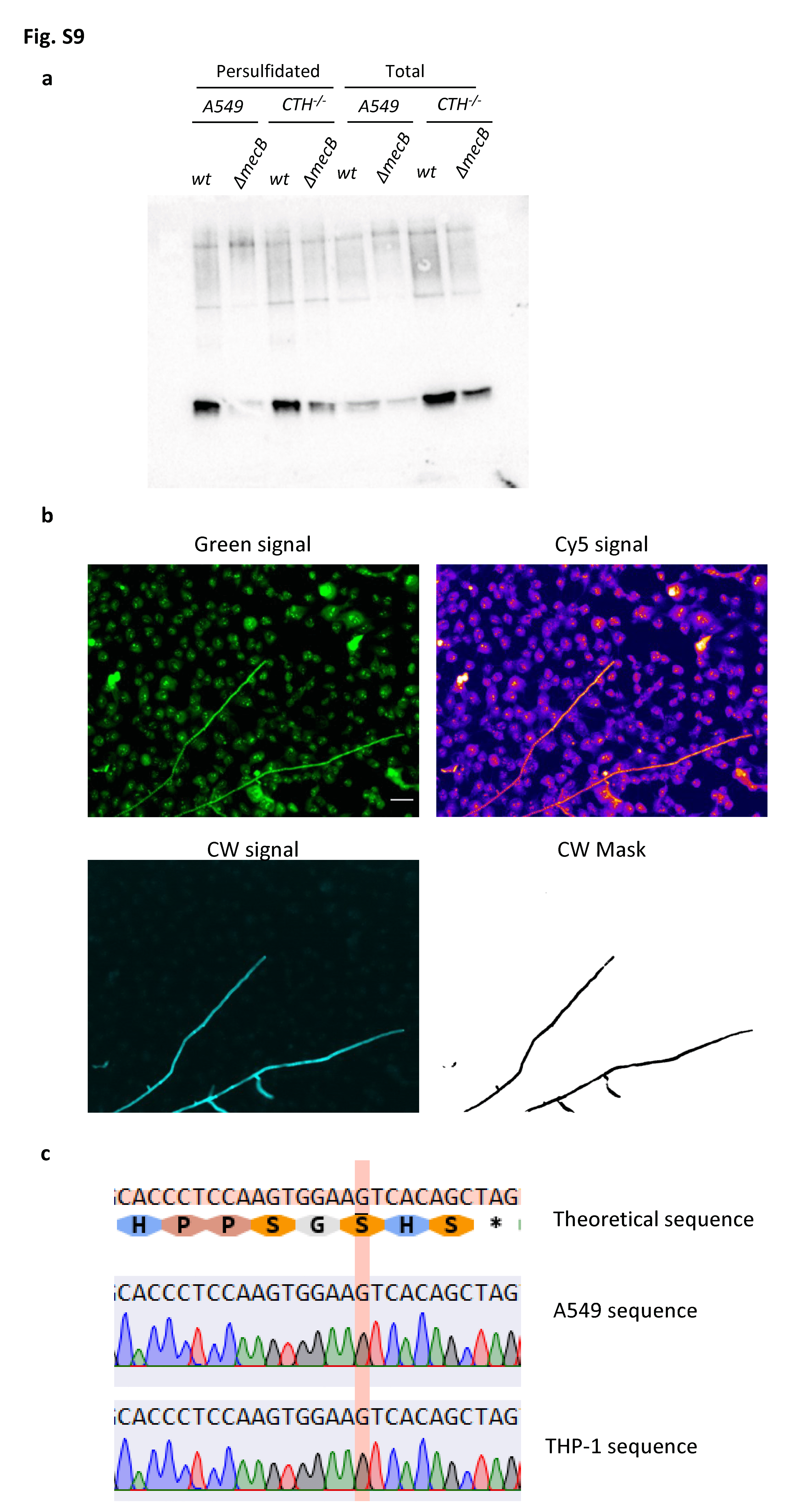

Supplement: S9 Fig — (a) Representative western blot of full protein lysate and persulfidated enriched fractions using an Aspf3 antiserum. (b) Representative images of the measurement of persulfidation levels in hyphae infecting an epithelial cell monolayer NBF-Cl labels persulfides, thiols, sulfenic acids, and amino groups; reaction with amino groups produces the green signal; therefore, it reflects the whole protein context and is used to normalize the persulfidation levels. The red signal is produced by the dimedone-Cy5 labeled probe, which selectively switches NBF-Cl in persulfide groups [20]. Calcofluor White dye specifically stains chitin in the fungal cell wall. This blue channel can be used to automatically segment hyphae (mask) and hence permits to measure the green and red signals exclusively from fungal cells in images containing mixed fungal and human cells. Scale bar = 30 μm. (c) Detail of the alignment of the wt theoretical CTH DNA sequence with the Sanger sequences from A549 and THP-1 PCR-amplified gDNAs. Both cell lines carry the original AGT codon for a serine (S) and therefore do not carry the rs1021737 SNP. CTH, cystathionine γ-lyase; NBF-Cl, 4-chloro-7-nitrobenzofurazan; wt, wild-type. (TIF) [file pbio.3001247.s009.tif]
