# Supplementary material for: Fungal and host protein persulfidation are functionally correlated and modulate both virulence and antifungal response
Source: PLoS Biol. 2021 Jun 1;19(6):e3001247. doi: 10.1371/journal.pbio.3001247 (PMC8168846; doi:10.1371/journal.pbio.3001247)
Supplement: S1 Table — The range of concentrations tested for each antifungal is depicted in brackets (mg/L). The MIC value of each drug was read at 24 and 48 hours after inoculation, and the calculated values (mg/L) are shown in the table. The assay was run using 2 biological replicates and 2 technical replicates. MIC, minimum inhibitory concentration. (DOCX) [file pbio.3001247.s010.docx]

|  | **Biological replica1** | | | | |  |
| --- | --- | --- | --- | --- | --- | --- |
|  | **AMB**  (16 - 0.03) | | **VRZ**  (8 - 0.015) | | **AND**  (16 - 0.03) | |
|  | **24 h** | **48 h** | **24 h** | **48 h** | **24 h** | **48 h** |
| wt (1) | 4 | 4 | 2 | 2 | 0,125 | 0,125 |
| wt (2) | 4 | 4 | 2 | 2 | 0,125 | 0,125 |
| *mecBΔ* (1) | 4 | 4 | 2 | 2 | 0,125 | 0,125 |
| *mecBΔ*  (2) | 4 | 4 | 2 | 2 | 0,125 | 0,125 |

|  | **Biological replica 2** | | | | |  |
| --- | --- | --- | --- | --- | --- | --- |
|  | **AMB**  (16 - 0.03) | | **VRZ**  (8 - 0.015) | | **AND**  (16 - 0.03) | |
| Time | **24 h** | **48 h** | **24 h** | **48 h** | **24 h** | **48 h** |
| wt (1) | 2 | 4 | 2 | 2 | 0,125 | 0,25 |
| wt (2) | 2 | 4 | 2 | 2 | 0,125 | 0,25 |
| *mecBΔ*  (1) | 2 | 4 | 2 | 2 | 0,125 | 0,125 |
| *mecBΔ* (2) | 2 | 4 | 2 | 2 | 0,125 | 0,125 |

**Table S1**

The minimum inhibitory concentration (MIC) of three drugs representative of each type of antifungal (AMB=Ambisome, polyene; VRC=Voriconazole, azole; AND= anidulafungin, echinocandin) were calculated for the *A. fumigatus* wild-type and *ΔmecB* strains. The range of concentrations tested for each antifungal is depicted in brackets (mg/L). The MIC value of each drug was read at 24 and 48 hours after inoculation and the calculated values (mg/L) are shown in the table. The assay was run using two biological replicates and two technical replicates
