# Supplementary material for: Fungal and host protein persulfidation are functionally correlated and modulate both virulence and antifungal response
Source: PLoS Biol. 2021 Jun 1;19(6):e3001247. doi: 10.1371/journal.pbio.3001247 (PMC8168846; doi:10.1371/journal.pbio.3001247)
Supplement: S3 Table — (DOCX) [file pbio.3001247.s012.docx]

| **Plasmid** | **Description** | **Reference** |
| --- | --- | --- |
| pUC19L | General cloning vector | Invitrogen |
| pSK529 | β-rec/*six* blaster module including hygromycin-B resistance cassette | Amich et at, 2013 |
| pSK530 | β-rec/*six* blaster module including pyrithiamine resistance cassette | Scott et al, 2019 |
| pJA4 | *A. fumigatus ∆mecA* replacement cassette: β‑rec/*six* blaster module from pSK529 (HygrB^R^) flanked by 5´and 3´*mecA* homology regions | Amich et at, 2016 |
| pJA26 | *A. fumigatus ∆mecB* replacement cassette: β‑rec/*six* blaster module from pSK530 (PtrA^R^) flanked by 5´and 3´*mecB* homology regions | This study |
| pJA47 | *A. fumigatus tetOFF_mecB* replacement cassette: tetOFF promoter module flanked by 1kb upstream 5’ region of *mecB* and *mecB* ORF as homology regions (PtrA^R^). | This study |
| pJA69 | *A. fumigatus tetOFF_mecA* replacement cassette: tetOFF promoter module flanked by 1kb upstream 5’ region of *mecA* and *mecA*ORF as homology regions (PtrA^R^). | This study |
| pJA57 | *A. fumigatus ∆mst* replacement cassette: β‑rec/*six* blaster module from pSK530 (PtrA^R^) flanked by 5´and 3´*mst* homology regions | This study |
| pJA103 | *A. fumigatus mecB ORF* under the control of its own promoter linked to a HygrB^R^ cassette | This study |
| pET-His6 TEV-LIC cloning vector (2B-T) | *E. coli* expression vector. T7 promoter. N-terminus His-tagg. | Gift from Scott Gradia (Addgene plasmid # 29666) |
| pJA103 | Human wild type CSE cloned in the pET-His6 expression vector | This study |
| pJA104 | Human CSE with SNP S403I cloned in the pET-His6 expression vector | This study |
| CTH CRISPR/Cas9 KO Plasmid (h2) | gRNA sequences direct the Cas9 protein to induce a site-specific double strand break in the in the genomic DNA *cth* gene | Santa Cruz Biotechnology |
| CTH HDR Plasmid (h2) | Homology-directed DNA repair (HDR) template, corresponding to the cut sites generated by the CTH CRISPR/Cas9 KO Plasmid (h2)., which inserts a puromycin resistance gene and an RFP (Red Fluorescent Protein) gene. | Santa Cruz Biotechnology |
