# Supplementary material for: Fungal and host protein persulfidation are functionally correlated and modulate both virulence and antifungal response
Source: PLoS Biol. 2021 Jun 1;19(6):e3001247. doi: 10.1371/journal.pbio.3001247 (PMC8168846; doi:10.1371/journal.pbio.3001247)
Supplement: Attachment — Submitted filename: Responses to Reviewers.docx [file pbio.3001247.s016.docx]

Reviewer #1:

The manuscript by Sueiro-Olivares et al describes roles for the posttranslational modification persulfidation in host-pathogen interactions and virulence of the human fungal pathogen Aspergillus fumigatus. The study tests the importance of both fungal and host persulfidation, by assaying mutants in fungal or mammalian cystatione gamma-lyase. In addition, several A. fumigatus proteins modified by persulfidation have been identified and the peroxiredoxin Aspf3 analysed further in several experiments. The conclusions are that, on the fungal side, persulfidation helps A. fumigatus to survive immune cell attack and drive disease, while on the host side, persulfidation assists immune cell responses (killing of fungal conidia and production of antifungal cytokines).

The study is interesting and the concept is novel - to my knowledge, this would be the first report of the importance of protein persulfidation in fungal pathogen-host interactions and disease. I have some concerns regarding the experiments and data interpretation, which I outline below.

We would like to thank the reviewer for taking time to review our manuscript and for the positive evaluation of its novelty and importance.

*       The authors show that the mecB mutant can utilise cysteine or methionine as a sole source of sulfur in vitro on plates (Fig S2d). This result is used to exclude effects of the mecB mutation on cysteine/methionine metabolism and ascribe phenotypes of the mutant exclusively to defective protein persulfidation. I wonder, however, if the metabolic role of mecB has some function in the survival of mutant conidia in macrophages, given the challenging nutritional environment intracellularly in phagocytes. At the very least, some discussion is warranted on this point.

We agree with the reviewer that, even if we believe the metabolic role of MecB is unlikely to explain the higher susceptibility to killing, our results do not completely rule out this possibility. Accordingly, we now mention this point in the manuscript (Lines 208-212)

*       The differences in survival between wild type and mecB mutant conidia in response to immune cells are small (for example 28 vs 16% in mouse macrophages and 14 versus 10% in human). I wonder if this small difference can be physiologically relevant. In other words, both wild type and mecB conidia survive to a substantial extent upon immune cell interactions. A similar small effect is seen for the CTH mutation on the ability of alveolar macrophages to kill Aspergillus conidia (Fig 3). The authors should consider discussing these points.

We again agree with the reviewer. It is true that the differences are small, but they are mathematically significant. We believe that these reductions are likely to be biologically significant, as supported by the facts that *A. fumigatus* *ΔmecB* has a decreased virulence in two murine models of invasive pulmonary aspergillosis and, concomitantly, that the SNP in human CTH is associated with higher incidence of aspergillosis. However, we acknowledge these effects on fungal virulence and risk of infection cannot be exclusively ascribed to the reductions in killing. Accordingly, we have now added some discussion to clarify that we propose they are the result of the combined effect of the altered activity of many proteins, affecting a variety of relevant processes, the mechanisms of which will need to be investigated in the future (Lines 399-402).

*       Western blots of Aspf3 used to determined persulfidated versus total protein should be improved. In particular, the Aspf3 signal in lysates is over-exposed (Fig S3a, S8a) and bands strangely shaped, which would make quantification difficult. It would also be advisable to show full Western blots (as this is supplemental data) and controls for specificity of the antibody.

We thank the reviewer for raising this point. We have repeated most of the Western-blots of the manuscript and show now full blots of better quality (no signal is overexposed, as confirmed by negative software detection of saturated pixels in the image). We have included the *Δaspf3* strain as the control of antibody specificity. Importantly, quantification of persulfidated Aspf3 in aspergillus extracts with the new blots show even a lower degree of persulfidation in *ΔmecB* compared with wild-type.

(one representative blot per experiment is shown in the correspondent Supplementary Figure. All gels and blots performed in the study are shown in the file Gels&Blots)

*       Fig S3c: Monomer/dimer quantification of Aspf3: there are multiple or smeared bands where presumably the monomer should be; it is therefore not clear what exactly was quantified. Bands used for quantification could be indicated and full gels (not cropped) shown, with controls for antibody specificity.

We thank the reviewer for mentioning this important matter. We have repeated these Western-blots using Acetamide to stabilize the monomers. We have also included *Δaspf3* as the control of antibody specificity. This has significantly improved the detection of dimers and monomers and thus the rigor of the quantification. Importantly, the ratio of Aspf3 monomers in *ΔmecB* is still clearly and significantly higher in the presence of H_2_O_2_, so our previous conclusions remains true (Figure S4c and Gels&Blots).

*       Statistics should be calculated using data points from independent biological repeats of the experiments. It is not clear to me that has been done in all cases. For example, Fig 1f - percentage of dead conidia: n=3 according to the figure legend, but many data points are plotted. I assume these data points are from the 3 technical repeats, 6 photos taken per well, as described in the legend. However, these data points are not all independent of each other  - it does not make much sense to do statistics on data obtained from different microscopy fields of the same wells or technical repeats  (which control for technical errors, but not for biological reproducibility of the data). Several other figures suffer from the same problem.
We are very thankful to the reviewer for this important correction. The reviewer is absolutely right, and we were presenting the data and running some of the statistical analyses incorrectly. We have now calculated one single value for each of the technical replicates (cumulative for the photos of each well), averaged the 3 technical replicates and thus used only one value per biological replicate. Encouragingly, even if the *n* has obviously decreased (to 3), the consolidation of the data has reduced the SD dramatically, and as a result the *P*-value has decreased in many cases. All the correspondent *P*-values have been updated in the text. The significance previously described and therefore the conclusions reached in the study have not changed.

Reviewer #2:
Summary
In this paper, authors enquired about the role H2S-mediated cysteine persulfidation has in the adaptation of Aspergillus fumigatus to its host and reciprocally the importance of the H2S host response in the defense against the pathogen. They conclude that persulfidation is important for both the pathogen and the host.

General comments
The question raised in this study is novel, important, and timely, as new tools are now available to monitor cysteine residues persulfidation. The paper unfortunately suffers from a poor organization and very casual presentation and writing, with many details missing in the text and figure legends, lack of explanations for many of the specific experimental approaches, which makes the reading very difficult and also forces the reader to dig into the methods. Several of the results presented are convincing, in particular le monitoring of persulfidation levels, and killing assays. Other, as described below are not convincing, or lack controls. Overall the conclusions of this study will be important, provided it is better supported by the data and presentation. The title does not reflects what is shown in the paper, with regards to the word interdependency, as not real interdependency is shown, as developed below, and with the wording disease severity, since authors were not able to reproduce a pulmonary aspergillosis in mice, at least with regards to the CTH-/- background (the explanation for their failure is not understandable), and the data from human cannot be taken as statistically significant (this part suffers from a very poor redaction, which makes it very hard to read).

We would like to thank the reviewer for taking the time to review our work and for the critical analysis made.

Addressing the reviewer criticism, we have made efforts to improve the clarity of our writing in the mentioned sections. We would like to highlight that this is a Discovery Report, which is a type of short article that describes early research outputs. Therefore, the length of the manuscript is limited and we cannot give all the technical details in the text (all are in M&M) nor elaborate too much on the results. Still, we have made our best to clarify the points that the reviewer criticized.

We have changed the title and we believe it now accurately reflects what is shown in the paper.

We now explain why we could not assay survival experiments with CTH-/- mice in more detail (Lines 305-311)

The human data of predisposition to IPA and MDMs cytokine production have been subjected to rigorous statistical analysis and is indeed significant. We acknowledge that persulfidation response in MDMs is not statistically significant, and state now in the text that “Additional experiments with more donors will be required to validate this result and understand the underlying mechanism” (Lines 246-247). However, we believe it is an interesting and valuable observation, which aligns well with all other results obtained in the course of the study.

Specific comments, major and minors
1. Failure of the generation of a mecAmecB mutant is interesting, but cannot be taken to say that persulfidation is a vital process in this fungus. This observation might rather be linked to the functions of the transsulfuration pathway in this sulfur assimilating fungus. Authors can attempt deleting one or the other gene with cover of the conditional expression of the other to prove their case
Addressing the reviewer’s comment, we have constructed strains using the TetOFF conditional promoter. However, as described in results (Lines 126-135 and Fig S1b&c) due to the high basal expression from the tet system, gene expression was not significantly lower in restrictive conditions compared with the wild-type transcription level. Therefore, and regrettably, we could not prove our point as suggested by the reviewer. We have also added a short paragraph in the discussion section to highlight how the use of conditional promoters is not as powerful as the heterokaryon rescue technique to demonstrate gene essentiality (Lines 405-410).

We would like to mention that the synthetic lethality of *mecA* and *mecB* is unlikely to be due to the functions in the transsulfuration pathway. Firstly, in other investigations we have constructed several viable double mutants in the pathway (*ΔcysDΔcysB*, *ΔcysBΔmecA* and *ΔcysDΔmetG*), which suggests that blocking the pathway in different parts does not prevent viability. Secondly, given our experience with the transsulfuration pathway we know that the provided sulfur source is important. Accordingly, we attempted to construct the double mutant on various single and combined sulfur sources (sulfate, cysteine and/or methionine). Providing both cysteine and methionine should reduce the urgency to interconvert them via the transsulfuration pathway, whilst intermediate products (as cystathionine) can still be produced from cysteine. Therefore, the implications of the genes in the transsulfuration pathway likely do not explain why we could not construct the double mutant.

Nevertheless, we agree with the reviewer that we cannot completely rule out the relevance of *mecA* and *mecB* function in the transsulfuration pathway. Accordingly, we have softened our statements to clearly state that we postulate that the genes are synthetic lethal likely because persulfidation cannot be abrogated (Lines 402-404).

2. Line 125 : what is fludioxonil, and why is it a glutathione-disturbing antifungal, and on which basis this is said?

We are very thankful to the reviewer for having realised of this mistake. Fludioxonil is a phenylpyrrol fungicide that inhibits the activity of a type III histidine kinase involved in glycerol biosynthesis and the osmotic stress response of filamentous fungi. The reason why we used it, which is now well explained in the text (Line 143), is because its fungicidal activity has been described to be enhanced when glutathione homeostasis is compromised (<https://doi.org/10.1111/j.1574-6968.2007.00682.x>).

3. S1b. Aspf3 and Pxr1 are shown to migrate at 25 and 35 kDa, respectively, which is surprising if indeed they represent two peroxiredoxins. What is their theorical size?

We are thankful with the reviewer for raising this concern. The theoretical size for PxrA (AFUB_065670) is 23.4 kDa and for Aspf3 (AFUA_4G08580) is 18.5 kDa. Therefore, the reviewer is right that they appear to have migrated at a size higher than expected. However, this is not completely unexpected as the Biotin-Thiol-Assay protocol employed in that experiment includes alkylation of the reactive -SH groups in cysteines with maleimide-PEG2-biotin, and therefore it is not surprising that the proteins migrate at higher sizes.

To avoid confusion and improve this result, we have repeated the protein identification experiment, but in this occasion we have utilized the dimedone switch protocol to enrich the persulfidation fraction, as we have demonstrated that it is more selective than the Biotin-Thiol-Assay. We have identified a total of 1313 proteins, including all those that we had identified in the previous experiment (Table S2 and Lines 165-171).

4. S3a. Authors should explain the rigor of the quantification of the persulfidation ratio. The only way how to measure this ratio is to compare the anti-Asp3 blot with the persulfidation fluorescence signal. The 3 blots used for this experiment should be shown.
We respectfully disagree with the reviewer. The only way to measure the ratio of persulfidated Aspf3 is to compare the enriched fraction with the total amount of Aspf3 protein (both specifically detected with the Aspf3 antiserum). The total levels of persulfidation (detected with the fluorescence signal) can vary due to changes in the persulfidation levels of other unrelated proteins, and therefore is not a valid value to calculate the aspf3 specific ratio.

We now show all blots used for this experiment in the file Gels&Blots.

Spore killing assays with macrophages: DmecB more sensitive to killing
Yes, exactly. This agrees with our hypothesis that lower persulfidation reduce *A. fumigatus* pathogenic potential.

5. Lane 159. Peroxiredoxins are usually very abundant enzymes, and it is hard to believe that a 20% decrease in its persulfidation will alter the activity of this enzyme in cells. The assay to measure Asp3 activity is totally non-specific, at least as it is described in the methods, and the differences of activity shown are not really impressive to conclude anything on this activity. Another assay that uses thioredoxin, thioredoxin reductase and NADPH, which specifies the activity of peroxiredoxins as thioredoxin-dependent thiol-based peroxidases should be done.

We thank the reviewer for this comment. Addressing it has improved our manuscript.

We would first like to mention that addressing reviewer1’s comment on the overexposure of the Western signals and its implication for the quantification, we have repeated this experiment. The new and more rigorous quantification with the new blots shows a higher decrease of Aspf3 persulfidation in *ΔmecB,* ~45% reduction (Line 180 and Fig. 1c). This higher reduction in Aspf3 persulfidation (which parallels the total level reduction in persulfidation of this mutant, Fig. 1a) explains better the observed decrease of Aspf3 activity.

As the reviewer suggested, in addition to the previously described assay, we have assayed a thioredoxin-dependent peroxidase activity assay. This new assay shows again a significant decrease of peroxiredoxin activity in *ΔmecB*, indicating that peroxiredoxin activity is reduced in low persulfidation (Lines 183-184 and 186 and Figure S4b)

6. How persulfidation affects peroxiredoxins is not well understood, if it indeed does anything. This question should first be addressed with recombinant proteins and enzyme assays. Whether persulfidation indeed protects from enzyme sulfinylation has indeed suggested recently, but these data must be confirmed. Anyhow, the blot of S3c cannot be evaluated: at best, it shows an increase in protein abundance, but not the band corresponding to the reduced (hyperoxidized monomer) and disulfide-linked dimer, which should be separated by at least the size of the monomer. How much H2O2 is used in this assay is not indicated. This experiment should be repeated by another method, for instance the use of an ab specific for the hyperoxidized form, or differential Cys residue alkylation.

We again thank the reviewer for this thoughtful comment.

Investigating how persulfidation affects peroxiredoxins is out of the scope of this manuscript, which focuses on the relevance of persulfidation for fungal virulence and host antifungal response. Nevertheless, our results support the previous suggestions that persulfidation protects peroxiredoxins from hyperoxidation (see below) and thus helps maintaining its activity under oxidative stress (previous point).

We have repeated and improved the Western-blot assay to detect Aspf3 dimers and hyperoxidized monomers, by optimising the conditions and using acetamide. The distinction of dimer and monomers in the new blots is much clearer, which has permitted a more rigorous and reliable quantification. We detect a clear and significant increase in the ratio of monomer to dimer ratio in *ΔmecB*, indicating a hyperoxidation of the protein (Fig S4c).

Furthermore, as suggested by the reviewer, we have used an antibody specific for the hyperoxidated form of persoxiredoxins (PrxSO3). Quantification of the hyperoxidated signal of all peroxiredoxins demonstrates that the oxidation level of these proteins increase upon H2O2 treatment and that the level of hyperoxidation is significantly higher in  *ΔmecB* compared with the wt strain (Lines 193-194 and Fig S4d).

7. Lane 178 states "in both cases", but which ones?

We have clarified that we mean that “Both cell lines killed *ΔmecB* conidia to a significantly higher level than wild-type conidia” (Lines 204-205)

8. The all paragraph starting at lane 195 should be re-written for the sake of clarity.

We have rewritten the paragraph and believe it is clearer now (starting in Line 226).

9. lane 198: explain what is a balanced persulfidation?

We mean that the levels of persulfidation are adequate to the cell needs. As this term seems to be confusing, we have substituted it for “adequately modulated” persulfidation levels (Line 228).

10: fig. 2b: on how many donors of this genotype was the experiment performed.

Regrettably, we could only get blood from one donor with this genotype for this experiment, this is mentioned in the figure legend (n=1). This is why we do not present any quantification. We acknowledge this result is not very strong on its own, but we believe it is interesting to present some data of persulfidation levels in human primary cells. Furthermore, it is supported by other results in the manuscript (e.g. the cytokine production).

Furthermore, the total lack of persulfidation response cannot intrinsic to the activity of CTH, but rather to some kind of defective signaling. The experiment also shows an highly significant increase in the basal levels of persulfidated proteins in the CTH SNP macrophages, which according to authors should be protective. Authors cannot throw in data without any explanations.

We acknowledge we cannot completely rule out some kind of deficient signalling. This is an intrinsic caveat of working with primary human cells, as they are not isogenic a perfect direct comparison is just impossible. Nevertheless, it is revealing that the cells which have a SNP in the gene encoding a protein which is major executer of persulfidation do not increase persulfidation levels as the “wild-type” genotype. We humbly believe that it is fair to presume a connection. In any case, we do not state in the text that the lack of response is directly caused by the activity of CTH, we just point out that in the TT genotype the response was blunted. This could be caused by CTH activity, but we agree with the reviewer that it could also be due to its implication in signalling. We have now included a sentence to clarify this (Lines 244-246).

Even if the basal level of persulfidation TT is higher, upon challenge with the fungus the level is clearly lower than in GG. According to our hypothesis, only the higher levels of persulfidation (correct physiological response upon challenge) would be protective.

We hope that our extended explanations are sufficient to understand the data we present.

11. Lane 211: the production of inflammatory cytokines comes here out of the blue, without any explanations of the possible link between this process and H2S production.

We now mention the reason why cytokine production may be affected in Lines 248-250.

12. Lane 214: how many individuals were studied? What was the study procedure: the full number of subjects, medical details… should be given?

Yes, of course, all this information is provided in Table 1.

What are the controls in these experiments.

We apologise, but we are not sure if we understand the reviewer´s question. The analysis detects whether the percentage of TT genotype is higher in the IPA group that in the non-IPA group, which serves as control of normal distribution of the SNP in the population. In order to clarify this, we have added the “Non-IPA” denomination to the control group in the table.

A table in the figure is not acceptable, and the figure totally lacks explanations of what is what.

We apologise, but due to the limitation in the number of figures and tables that can be presented in a Discovery Report short article, we must include this data within the figure to be able to show all our results. We would expect that the editorial office let us know if we need to accommodate the data in a different format.

This type of graph is the standard to present genotype associated incidence data. We have slightly modified its format and added more details in the figure legend aiming to facilitate its understanding.

13. Lane 216: the sentence is not understandable: any effect of what?

We clarify now that we did not detect any effect of the presence of the SNP in the donor compartment on the incidence of IPA in transplant recipients (Line 256).

14. Lanes 239-40: they killed at higher degree compared to what?

Added “than wild-type conidia” (Line 282).

15. The paragraph starting at lane 229 should be written again, for the sake of clarity

We have edited this paragraph and believe it is clearer now (starting in 271).

16. Lane 244: What are these mice: for clarity, start with one class/subject/genetic backround/treatment, genetic, treatment, and then go to the point; then do the other classes.

As we have edited the whole paragraph, we believe this analysis is clearer now (starting in Line 287).

17. What this result means?

The meaning of the result is better described in the new text

18. lane 269 what is the value indicated?

The value is described now as more or less percentage of detachment, which we hope is clearer (Lines 321 -324).

19. Lane 292: the hypothesis presented is not clear.

We have rephrased the sentence to present our hypothesis more clearly (Lines 346-348).

20. Lane 295: what is the meaning of "full"

We mean the total protein fraction. For clarity, we have substituted the word full for “total”

and edited the sentence (Line 349).

21. Fig 4a and S8a: as already asked, how is the quantification performed? It should compare the signal obtained by western blot on the precipitated protein, relative to the P-SS fluorescence signal. Furthermore, the protein WB is overloaded to be able to quantify it. It should be reloaded with less protein. The entire gel should be shown. In 4 a, the values for the Wt is 1.1: what this mean?

As we have explained above, we believe that the only way to measure the ratio of persulfidated Aspf3 is to compare the enriched fraction with the total amount of Aspf3 protein (both specifically detected with the Aspf3 antiserum). The levels of total protein persulfidation (detected with the fluorescence signal) can vary due to changes in the persulfidation levels of other unrelated proteins, and therefore is not a valid value to calculate the Aspf3 specific ratio.

As requested by reviewer 1, we have repeated the Western-blot using less protein of the total fraction, so the band is not overloaded. We have repeated the whole experiment and calculated the ratio of Aspf3 persulfidation again. As can be noticed in the figure (we show entire gels), newly calculated ratios of persulfidated Aspf3 are comparable to the previously obtained values, corroborating the conclusions that we have presented in the manuscript.

In the previous graph we were relating the mean of all values, including the wild-type in A549, to the median of the replicates of the control condition. As there was one outlier in one of the samples, the relative value was 1.132. We were showing the results in that format because we considered it takes more into account the variability of the control condition for the analysis. However, the fact that this reviewer asked about it demonstrates that this was difficult to understand. Therefore, we now relate the values of each experiment to the mean of the control condition, such that the relative value of the control is 1.

22. Lane 301: sentence unclear, and explain why it is important.

We have slightly edited the sentence to facilitate its understanding and to explain why it is important. This is because if MecB is the major responsible for the specific increase in persulfidation in response to host challenge, it is conceivably that MecB is the most relevant of the persulfidating proteins for fungal virulence (Lines 357-359).

23. That the level of host persulfidation influences the level of fungal persulfidation is okay, but the experiment proposed fail to answer why. Why the fungus adaptive response should be minored in the CTH-/- mice is not revealed.

We agree we do not give a final answer to the mechanism. Our experiment suggests that there is not direct transfer of produced H_2_S_2_ from the host to the fungus. Rather, it seems that the high antifungal potency of cells that can modulate their levels of persulfidation cause higher stress to the fungus, which needs to respond by increasing its own persulfidation levels. To what extent this adaptive response is important for pulmonary infection in mice will need to be tested in future investigations. Please, remember this is a Discovery Report and therefore it is not expected that all mechanisms are completely elucidated. We have added a paragraph at the of the section to state the conclusions we have reached in this manuscript and clarify that future research is needed to understand the mechanisms (Lines 379-383).

Reviewer #3:
This is an interesting, well-written manuscript about a reciprocal relationship of persulfidation between host and pathogen, using in vitro and animal experimental models of aspergillosis, and with clinical associations. A strength of the report is the manner which the problem has been addressed from multiple hypothesis-testing experimental angles.
We would like to thank the reviewer for taking the time to review our study and the positive comments about it.

COMMENTS
1. Abstract "… and predisposes ….". The clinical study about the CTH SNP is a correlation and not a proof of causality (as is correctly summarised on page 4, last paragraph). This should therefore more correctly say " …. and correlates with a predisposition …. "

Changed as suggested. Thank you for the correction.

2. As several in vitro studies rely on studies done using the human cell line THP-1, its CTH allelotype at the SNP location should be explicitly confirmed as wild type.
Also, although this is less important for the A549 human alveolar macrophage cell line studies as the test group was an intentional CTH knockdown, it would have been prudent also to check their genotype. I note from methods (p27) and Supplementary Figure S4 that the knockout status of A549 was confirmed by Western blot. Yet the line is referred to by its genotype "CTH-/-". Hence, confirm explicitly that the Western blot phenotype confirmed a molecularly-demonstrated knockout genotype.

We thank the reviewer for these important comments. The reviewer is absolutely right that it was important to confirm the CTH allelotype in THP-1 and A549. We have extracted DNA of both cell lines, amplified the region of interest by PCR and Sanger sequenced it. We can confirm that both lines are homologous for the wild-type GG genotype. This is now mentioned in Material and methods (Lines 685-695) and shown in Fig. S9c.

We added the sentence suggested by the reviewer to explicitly state that the Western blot phenotype confirmed a molecularly-demonstrated knockout genotype (Line 314 in results and 710 in M&M).

3. The animal experiments (Fig 1 g-h) appears to have been done once, with n=11 mice per group.  Hence displacement between the survival curves in time is highly vulnerable to inequalities in the inoculated dose and delivered inoculated dose of the control and test spore preparation. Were these validated by plating of inoculated volumes? Were these results consistent by replication in experiments using inoculae that were prepared completely independently of each other? At the moment, particularly if the 1g and 1h experiments were performed side-by-side as can't be excluded, there is the possibility that a single unintended difference in the final concentration of two spore suspensions resulting a larger inoculum concentration in the test mutant group could account for these survival curve differences.

We understand the reviewer’s concern and would like to explain these experiments in detail. Each experiment was indeed performed only once with n=11. However, the experiments were not performed side-by-side. We initially only assayed the leukopenic model and a few months later decided it would be interesting to confirm in a corticosteroid model. Furthermore, it is normal practise in our institution to always plate the prepared infection inocula to count the inoculated viable CFUs. In this case, we found that the viable counts were: 1) leukopenic experiment, 1.5 ×10^4^ wild-type and 1.45 ×10^4^ *ΔmecB* and 2) corticosteroid experiment, 1.33 ×10^6^ wild-type and 1.05 ×10^6^ *ΔmecB*. Therefore, in the leukopenic experiment the infectious inocula were very similar (although a bit lower than the 2 ×10^4^ expected, so we have corrected this value in material and methods, Line 808) and in the corticosteroid experiment the infectious dose of the mutant was higher than the wild-type control, but still caused less mortality.

Based on all the above, we believe that we can rule out an effect of the inocula on the observed difference in mortality.

4. The statement (line 216-18) dissociates the correlation between IPA incidence and donor SNP genotype, but localises it to the recipient genotype.  This prompted me to question whether the alveolar macrophage genotype (presumably donor derived) was relevant, and how the hypothesis/mechanistic picture accommodated this fact.

Alveolar macrophages are a complicated cell population. They are a very stable and long-lived population that can self-maintain for life ([10.1084/jem.20131199](https://doi.org/10.1084/jem.20131199) / [10.1242/dev.129122](https://dx.doi.org/10.1242%2Fdev.129122)). Furthermore, they are quite resistant to conditioning treatments (10.1080/01902140802022518 / [10.1016/j.immuni.2013.04.004](https://doi.org/10.1016/j.immuni.2013.04.004) / [10.1165/rcmb.2007-0224RC](https://doi.org/10.1165/rcmb.2007-0224rc) / 10.1128/mBio.02752-19) which means that a significant part of the AMs present in the lungs of transplant recipients will be recipient derived for a long period of time. We have added a sentence to clarify this point and included the mentioned references to support the consideration of AMs as recipient-derived (Lines 271-276).

The interpretation/discussion was undisciplined in regard to the distinction between macrophage and alveolar cell genotype (for example line 354-5 focuses on macrophage, non alveolar epithelial cell, genotype in this context). This point is also blurred in lines 33-34 of the abstract ( "correct levels of persulfidation are required for optimal antifungal activity of lung-resident host cells"). I believe that greater clarity regarding what the hypothesis is and what the data show is required on this point.
We thank the reviewer for raising this important issue, which was clearly not well explained. We have now included new text to facilitate understanding of the interpretation and discussion of our data.

In conjunction with the previous point, we have made clear that AMs can be considered at least partially as recipient derived for long periods of time (Lines 271-276). Accordingly, we mention in the abstract that lung-resident host cells are recipients’ (Line 38). We have included a paragraph at the end of the results section to clearly state the conclusions reached in this study (Lines 379-383) Finally, we have included more discussion to explain why we believe the SNP on the donor side does not correlate with higher predisposition to IPA and more clearly explain our hypothesis why the SNP on the recipient side does (Lines 426-433).

MINOR COMMENTS
Page 4: " ….. including an estimated 50,000 cases of lethal invasive pulmonary aspergillosis (IPA) (15)." I was interested in these numbers and so looked up reference 15. Actually, it mentions only invasive aspergillosis, not invasive pulmonary aspergillosis, and it does not provide a mortality rate. Although it most case will be pulmonary, and although it will often be lethal, this reference does not establish either of those points.

We thank the reviewer for the correction of these important details. According to those, we have eliminated “pulmonary” and substituted lethal for “life-threatening”. We have added another, more recent, reference to mention the global estimate of the incidence of IA and its associated mortality rate (Lines 79-81).

Page 8: "…..enzymes'…." the apostrophe should precede the "s" as it is only one enzyme being talked about in the possessive

Corrected (Line 190).

Table S1. The abbreviations in the headings are not defined within the table or elsewhere; while I could guess some, this should not be necessary. What are the bracketed figures in the line R3? What do the numbers within the cells of the table mean?

We apologize for the poor presentation of the data contained in this table. We have now improved the table and added a legend to explain it in detail

Lines 331-332 "…the consequences …. was not investigated …." (should be "were").

Corrected (Line 393).
