# Supplementary material for: Fungal and host protein persulfidation are functionally correlated and modulate both virulence and antifungal response
Source: PLoS Biol. 2021 Jun 1;19(6):e3001247. doi: 10.1371/journal.pbio.3001247 (PMC8168846; doi:10.1371/journal.pbio.3001247)
Supplement: Attachment — Submitted filename: Responses to Reviewers.docx [file pbio.3001247.s017.docx]

Reviewer #2:
[identifies himself as Michel Toledano]

Summary
This is a resubmitted manuscript on a host-pathogen reciprocal relationship linking the levels of general persulfidation in A fumigatus and lung epithelial cells, as explored by the use of cells with mutations in CSE, one of the main enzyme for H2S production. The data show that fungi with decreased persulfidation have a decreased pathogenic potential in mice/resistance to killing by macrophages in vitro; reciprocally murine or human cells with a CSE knockout have defect in fungi killing and in mounting an inflammatory response. Further, the data show a positive correlation between the level of persulfidation of host cells and the persulfidation response in fungi. Lastly, authors identify in human databases a SNP in CSE, and show it decreases CSE enzymatic activity (H2S production) and its presence correlates with a higher incidence of invasive aspergillosis in immunocompromised transplant recipients.

General comments
The efforts of authors in answering and improving the manuscript is fully acknowledged. The criticisms made in the previews review have been handled satisfactorily, with one exception that relates to the part on Asp f3, as indicated below. The paper is much improved, and as previously acknowledged, significant for its general message.

Dear Dr Toledano, we would like to thank you for revising our paper again, for taking into account the great effort we have made and considering that the paper has improved.

Specific comments
1. The synthetic lethality resulting from MicA and MicB gene deletion should be pursued, because if indeed persulfidation is essential for A fumigatus viability, why is it so should provide a significant clue to the physiological relevance and mode of action of H2S. However, as stated before, there is no proof whatsoever that lethality is the result of decreased H2S production, especially under laboratory conditions with no stress imposition. Therefore, even as a postulate, it is not justified by data.

We agree with Dr Toledano that this is a very interesting aspect of the research and will aim to pursue it in the future. However, at this moment there are simply no tools in *Aspergillus* to do so. We have attempted to use two different repressible promoters (in addition to the TetOFF we also made a slight attempt with the XylP promoter, with similar result) and they do not work satisfactorily. We are currently working in the laboratory to develop optimised genetic tools, but that is a whole new project which will take months or years to be ready.

According to the Dr Toledano’s criticism, we have eliminated our postulate that synthetic lethality might be due to a decrease in persulfidation from the results section (where data is presented). However, we mention that this is our hypothesis in the discussion. Future experiments will determine whether our hypothesis is correct or not, but we believe it is fair to propose it in our discussion of the results. We have edited this section in the discussion to clarify that this is our current working hypothesis (Lines 419-422 and 430-436).

2. There is still a problem with data pertaining to Aspf f3.  That enzyme persulfidation decreases in the MecB mutant is convincing. The problem is again on the activity assays and on the monitoring of Asp f3 sulfinylation. On the assays: the first assay is totally unspecific to Asp f3: authors should thus present the data by saying that inactivating CSE decreases the ability of this fungus to degrade the H2O2 present in the cell culture, without referring to Asp f3, the defective persulfidation of which would cause this scavenging defect. Regarding the other assay, this is again an in vivo assay that requires many biochemical steps through which persulfidation might be lost. Although it shows a decreased activity, these in vivo activity assays are not really reliable: the only way to answer the question raised is by performing in vitro assays with recombinant proteins. On sulfinylation: sensitivity to this PTM is an attribute carried by some, but not all Prx family members,
and there is no indication that Asp f3 carries this attribute. In fact, as indicated by the report of Hillmann et al, Asp f3 is homologous to the Prx human variant Prx5 and functionally homologous to the yeast isotype logue Pxr5 , which are enzymes that are both known for not undergoing sulfinylation. Further, the blot of S4D does not show any detectable increase in the sulfinylation signal between untreated and H2O2 exposed Wt cells, which would indicate that this enzyme is not sensitive to hyperoxidation. The suggestion here would be to remove this all part as it significantly depart by its weakness from the quality of the manuscript, especially if it is not in the paper scope.

We would like to thank Dr Toledano for his detailed analysis of this section of the manuscript. Please, allow us responding to the concerns separately:

1. In respect to the enzymatic assays:

We agree that the first assay is unspecific and therefore we cannot ascribe the reduction in H_2_O_2_ detoxification activity to exclusively to peroxiredoxins. However, the second assay is specific to peroxiredoxins; therefore, we now state in the text that in low persulfidation the level of H_2_O_2_ detoxifying activity is affected, including peroxiredoxin activity, without mentioning Aspf3 (lines 190-191). However, we disagree that the thioredoxin-dependent in vivo assay is not reliable to detect reduction in activity due to decreased persulfidation levels. This assay was previously used with cell lysates (10.1016/j.ab.2004.12.008), demonstrating that it works fine with homogenates. Here, we could detect decreased activity in the low persulfidating mutant, indicating that in low persulfidation the overall peroxiredoxin activity is reduced. A reduced peroxiredoxin activity in low persulfidation agrees with a previous study that reported how persulfidation protects peroxiredoxins from irreversible oxidation, therefore maintaining their function (10.1126/sciadv.aax8358), we know cite this study in the paper (lines 191-193).

Therefore, even if we agree that the most correct way to show a specific decrease of Aspf3 activity (or of other peroxiredoxins) would be to use recombinant proteins, we believe that with our results we can confidently conclude that in low persulfidation the overall peroxiredoxin activity is reduced.

It is worth mentioning that a proper in vitro analysis of specific peroxiredoxin functions could constitute a whole paper for itself and it is out of the scope of this study.

2. In respect to sulfynilation of Aspf3:

We would like to thank Dr Toledano for highlighting this matter. We have further investigated the possibility that Aspf3 could undergo hyperoxidation in high H_2_O_2_. We labelled sulfynilated proteins with the Biotinylated-DiaAlk probe (Akter et al 2018, https://doi.org/10.1038/s41589-018-0116-2), immunoprecipitated Aspf3 and tried to detect its binding to the biotinylated probe. However, we did not detect binding of the probe to Aspf3 (Fig S4f), indicating that the protein is not hyperoxidated, as Dr Toledano pointed out. We show this result in the manuscript as we believe it is good to report to the community that Aspf3 does not undergo hyperoxidation (lines 199-203).

To further characterise the consequences of low persulfidation, we have measured total levels of protein sulfenylation (Fig. S4d) and sulfinylation (Fig. S4e) and found that in the presence of H_2_O_2_ *ΔmecB* has higher levels of oxidation than the wild-type strain (lines 197-199).

Furthermore, we have made a quantification of the PrxSO3 signal of one of the bands previously shown in the blot (~20 kDa). As can be seen in the table below, the level of hyperoxidation of this band seems to be increased in the *ΔmecB* mutant upon H_2_O_2_ treatment. Nevertheless, as requested by Dr Toledano, we have eliminated this result from the paper. We do not know the identity of this peroxiredoxin (we do not have antibodies against other peroxiredoxins) and performing a characterisation of its hyperoxidation and activity is out of the scope of this study.

|  |  |
| --- | --- |
| WT | 1 |
| ΔMecB | 0.821021 |
| ΔAspf3 | 0.647329 |
| WT+ H2O2 | 0.9852 |
| ΔMecB+H2O2 | 1.159 |
| ΔAspf3+H2O2 | 0.675673 |

With all our results we believe that our current conclusions are well supported: 1) in low persulfidation there are higher levels of protein oxidation, 2) due to an impacted H_2_O_2_ detoxifying activity, including overall reduced peroxiredoxin activity. This demonstrates that persulfidation is relevant for a process that is known to be important for fungal virulence, which was the aim of this section.

We would like to thank Dr Toledano again for helping to avoid inaccuracies in this section. We believe that we now have made the correct conclusions without any unproven assumptions. We hope Dr Toledano agrees.

3. What is the difference between the experiments shown in Fig. 1 h, I and the one intended with CTH-/- mice, but not successful? If it is the same what did it work in the former but not in the latter case?

The difference is the mouse line utilised: CD1 in figure 1 (the mouse line we routinely use for *A. fumigatus* infections) and C57BL/6 (background line where the CTH^-/-^ was generated) in the latter. As explained and cited in the pertinent paragraph, there are anatomical differences in the airways of these two mouse lines, which are known to influence particle deposition. The fact that for C57BL/6, a high percentage of mice develop balance problems suggest that there is a high deposition of spores in the sinus, rather than in the lungs. This causes that, when the fungus grows, it invades the inner ear and maybe even the brain and causes imbalance and neurological symptoms. This is something known to happen sometimes (we obtain ~1% of this in the CD1 line), but not expectable with such a high frequency. This same effect is causing significant problems in Prof Beilhack’s laboratory, in which many murine knock-out lines are constructed in the C57BL/6 background. Consultation with other laboratories has revealed that many groups experience similar problems, but it seems it has not been reported before. Therefore, it will actually be valuable for the community to have this piece of information published.

In our case this completely invalidates the experiment. Firstly, because the mice that develop imbalance symptoms must be immediately sacrificed (therefore with such high numbers the survival curves are compromised). Secondly because we have shown a role of CTH for antifungal defence in lung resident cells; therefore, if there is extrapulmonary deposition of fungal spores we cannot evaluate the role of CTH for susceptibility to infection.

Reviewer #3:

I have re-read the manuscript and noted the responses to my previous comments. Most are adequately addressed – thank you for the additional explanations.  I have also noted the other reviewer comments and the responses to them.

We would like to thank the reviewer for reading the manuscript again.

However, regarding my previous point 3, which questioned the degree of replication of the in vivo experiments, there are still concerns. The authors have indicated that each survival cohort has been run only once, and now revealed that also they were not run concurrently. By providing more information about back-plated determinations of CFU inocula for infectious dose verification, they argue that differences in inoculated doses between groups are not relevant. This scenario is problematic overall because:

(1) the lack of experimental replication means it is not established that the variation due to the test intervention (genotype) is greater than the variation between running the experiment independently on different days (even despite n=11 mice per group).  The point being evaluated is the difference in the survival curves between groups; while n=11 within the groups provides some power to discern/resolve differences, when it reflects the course of single cohorts in a single experiment, it does not establish reproducibility.

(2) for the corticosteroid experiment, the stated inocula doses were "1.33×10^6 wild-type and 1.05 ×10^6 ΔmecB", indicating that the WT dose was 30% greater than the mutant dose. Yet following it is stated that "in the corticosteroid experiment the infectious dose of the mutant was higher than the wild-type control, but still caused less mortality", which is not correct based on the doses given, and if the numbers are correct negates the argument being presented in favour of a true mutant-dependent reduction in mortality effect.

To deal firstly with the point number (2) of the reviewer, we would like to apologise for the typographical mistake in our previous response, which inverted the identities of the inocula. The correct values are: 1.33×10^6 for *ΔmecB* and 1.05 ×10^6 for wild-type. Therefore, our reasoning that “the infectious dose of the mutant was higher than the wild-type control, but still caused less mortality” is true and supports the argument of a mutant-dependent effect.

With respect to the reviewer’s concerns about reproducibility of virulence data we hope that, coupled with the above correction of the typographical error, the following response will allay those concerns.

Investigations of *A. fumigatus* virulence routinely survey susceptibility in two different models of host susceptibility, both of which emulate human risk factors for invasive lung disease. Reproducibility across two different models, independently performed in outbred mouse lines where genetic heterogeneity is expected, provides good evidence that virulence factors have relevance across a diversity of genetic and pathological settings in which aspergilloses occur. Moreover, this convention satisfies the expectation that lowering standardization in the execution of experiments (for instance, by performing multilaboratory studies by using different animal strains and sexes, or by diversifying housing conditions) can boost the external validity of research results [1].

Accordingly, we have investigated *ΔmecB* virulence in two routinely used murine models of pulmonary aspergillosis, which were performed independently (not concurrently). In both experiments a significant decrease in virulence of the *ΔmecB* mutant was detected. Therefore, we have obtained independent evidence for a reduction of *ΔmecB* virulence from two independent experiments. Furthermore, we use CD1 outbred mice, which are genetically diverse and thus produce a variable response to infection within each group, which is proposed to provide more robust conclusions and maximize reproducibility [2,3]

1. Bert B, Heinl C, Chmielewska J, Schwarz F, Grune B, Hensel A, et al. (2019) Refining animal research: The Animal Study Registry. PLoS Biol 17(10): e3000463. https://doi.org/10.1371/journal.pbio.3000463
2. Enriquez, J., Mims, B.M.D., Trasti, S. *et al.* Genomic, microbial and environmental standardization in animal experimentation limiting immunological discovery. *BMC Immunol* **21,** 50 (2020). <https://doi.org/10.1186/s12865-020-00380-x>
3. Tuttle, A.H., Philip, V.M., Chesler, E.J. *et al.* Comparing phenotypic variation between inbred and outbred mice. *Nat Methods* **15,** 994–996 (2018). https://doi.org/10.1038/s41592-018-0224-7

It is important to remark that PLOS Biology publications do not routinely dictate independent repetition of infection models as a standard requirement for demonstrating reduction of virulence of a mutant strain. Indeed, repetition of the experiment was not required in a variety of murine models of infection included in numerous published PLOS Biology papers, when looking at either survival [4-8] or at other virulence-related traits [9-15]. Similarly, repetition of infection experiments is not an indispensable requirement in peer-reviewed literature to evaluate the virulence of *A. fumigatus* mutants, as exemplified with references [16-21].

4. Xu W, Solis NV, Ehrlich RL, Woolford CA, Filler SG, Mitchell AP (2015) Activation and Alliance of Regulatory Pathways in C. albicans during Mammalian Infection. PLoS Biol 13(2): e1002076. <https://doi.org/10.1371/journal.pbio.1002076>
5. Tao L, Du H, Guan G, Dai Y, Nobile CJ, Liang W, et al. (2014) Discovery of a “White-Gray-Opaque” Tristable Phenotypic Switching System in Candida albicans: Roles of Non-genetic Diversity in Host Adaptation. PLoS Biol 12(4): e1001830. <https://doi.org/10.1371/journal.pbio.1001830>
6. Zhao J, Yu X, Zhu M, Kang H, Ma J, Wu M, et al. (2016) Structural and Molecular Mechanism of CdpR Involved in Quorum-Sensing and Bacterial Virulence in Pseudomonas aeruginosa. PLoS Biol 14(4): e1002449. <https://doi.org/10.1371/journal.pbio.1002449>
7. Norris MH, Kirpich A, Bluhm AP, Zincke D, Hadfield T, Ponciano JM, et al. (2020) Convergent evolution of diverse Bacillus anthracis outbreak strains toward altered surface oligosaccharides that modulate anthrax pathogenesis. PLoS Biol 18(12): e3001052. <https://doi.org/10.1371/journal.pbio.3001052>
8. Tsugawa H, Kabe Y, Kanai A, Sugiura Y, Hida S, Taniguchi S, et al. (2020) Short-chain fatty acids bind to apoptosis-associated speck-like protein to activate inflammasome complex to prevent Salmonella infection. PLoS Biol 18(9): e3000813. <https://doi.org/10.1371/journal.pbio.3000813>
9. Kaiser P, Regoes RR, Dolowschiak T, Wotzka SY, Lengefeld J, Slack E, et al. (2014) Cecum Lymph Node Dendritic Cells Harbor Slow-Growing Bacteria Phenotypically Tolerant to Antibiotic Treatment. PLoS Biol 12(2): e1001793. <https://doi.org/10.1371/journal.pbio.1001793>
10. Stecher B, Robbiani R, Walker AW, Westendorf AM, Barthel M, Kremer M, et al. (2007) Salmonella enterica Serovar Typhimurium Exploits Inflammation to Compete with the Intestinal Microbiota. PLoS Biol 5(10): e244. <https://doi.org/10.1371/journal.pbio.0050244>
11. Gao B, Vorwerk H, Huber C, Lara-Tejero M, Mohr J, Goodman AL, et al. (2017) Metabolic and fitness determinants for in vitro growth and intestinal colonization of the bacterial pathogen Campylobacter jejuni. PLoS Biol 15(5): e2001390. <https://doi.org/10.1371/journal.pbio.2001390>
12. Barclay VC, Sim D, Chan BHK, Nell LA, Rabaa MA, Bell AS, et al. (2012) The Evolutionary Consequences of Blood-Stage Vaccination on the Rodent Malaria Plasmodium chabaudi. PLoS Biol 10(7): e1001368. <https://doi.org/10.1371/journal.pbio.1001368>
13. Mackinnon MJ, Read AF (2004) Immunity Promotes Virulence Evolution in a Malaria Model. PLoS Biol 2(9): e230. <https://doi.org/10.1371/journal.pbio.0020230>
14. Qi Y-J, Jiao Y-L, Chen P, Kong J-Y, Gu B-L, Liu K, et al. (2020) Porphyromonas gingivalis promotes progression of esophageal squamous cell cancer via TGFβ-dependent Smad/YAP/TAZ signaling. PLoS Biol 18(9): e3000825. <https://doi.org/10.1371/journal.pbio.3000825>
15. Barriales D, Martín-Ruiz I, Carreras-González A, Montesinos-Robledo M, Azkargorta M, Iloro I, et al. (2021) Borrelia burgdorferi infection induces long-term memory-like responses in macrophages with tissue-wide consequences in the heart. PLoS Biol 19(1): e3001062. <https://doi.org/10.1371/journal.pbio.3001062>
16. Kowalski, C.H., Kerkaert, J.D., Liu, KW. *et al.* Fungal biofilm morphology impacts hypoxia fitness and disease progression. *Nat Microbiol* **4,** 2430–2441 (2019). <https://doi.org/10.1038/s41564-019-0558-7>
17. Wang, X., Caffrey-Carr, A. K., Liu, K., Espinosa, V., Croteau, W., Dhingra, S., et al. (2020). MDA5 Is an Essential Sensor of a Pathogen-Associated Molecular Pattern Associated with Vitality That Is Necessary for Host Resistance against *Aspergillus fumigatus. The Journal of Immunology, ji2000802.* doi:10.4049/jimmunol.2000802
18. Voltersen V, Blango MG, Herrmann S, Schmidt F, Heinekamp T, et al. 2018. Proteome analysis reveals the conidial surface protein CcpA essential for virulence of the pathogenic fungus Aspergillus fumigatus. mBio 9:e01557-18. <https://doi.org/10.1128/mBio.01557-18>
19. Furukawa, T., van Rhijn, N., Fraczek, M. *et al.* The negative cofactor 2 complex is a key regulator of drug resistance in *Aspergillus fumigatus*. *Nat Commun* **11,** 427 (2020). <https://doi.org/10.1038/s41467-019-14191-1>
20. Weichert M, Guirao-Abad J, Aimanianda V, Krishnan K, Grisham C, et al. 2020. Functional coupling between the unfolded protein response and endoplasmic reticulum/Golgi Ca^2+^-ATPases promotes stress tolerance, cell wall biosynthesis, and virulence of Aspergillus fumigatus. mBio 11:e01060-20. <https://doi.org/10.1128/mBio.01060-20>.
21. Hillmann, F., Bagramyan, K., Straßburger, M. et al*.* The Crystal Structure of Peroxiredoxin Asp f3 Provides Mechanistic Insight into Oxidative Stress Resistance and Virulence of *Aspergillus fumigatus*. *Sci Rep* **6,** 33396 (2016). https://doi.org/10.1038/srep33396

Considering all of the above points, we believe that repeating the infections experiments is 1) not scientifically required to demonstrate a reduction in *ΔmecB* virulence and consequently 2) not ethically justifiable, as would breach the regulations of the 3Rs of mice. Indeed, in an informal discussion about the possibility of repeating the experiments with the Project Licence Holder, the Manchester Named Compliance Officer (NCO) and the Named Animal Care and Welfare Officer (NACWO) we were informed that this would not be acceptable.

(3) The methods section lines (807-809) should provide the verified inocula, given as viable spores (not just spores), as it was established by back-plating (not current revealed in the methods section), and the difference between 1.33 and 1.05 is approx. 31% and should not be stated as being 1 ×10^6 for both groups.

We agree with the reviewer it is more correct to report the exact infection doses. Accordingly, we have added the details of the viable counts in the methods section (lines 885-887).

(4) It is not clear if the inocula sizes were almost 100-fold different in the leukopenic and corticosteroid models for an intended reason such as prior knowledge of the appropriate dose in WT animals (if so, provide citation), or if it was the case just by chance, perhaps because the experiments were not done concurrently.

It is well established and widely known that murine (and human) susceptibility to pulmonary aspergillosis depends on the degree of immunosuppression. Leukopenic mice are deeply immunosuppressed and therefore a low inoculum causes lethal infection. The degree of suppression is lower in corticosteroid treated animals and therefore higher infection doses are required. We have included a reference for a recent review which provides ample information about these models (lines 225-226).

Incidentally, I noted that the competing interest statement in the paper itself (none to declare) is different from that in the front-end material (where one author declares a competing interest).

We would like to than the reviewer for noticing this error. We have updated the statement in the paper (lines 988-990).
