# Supplementary material for: Fungal and host protein persulfidation are functionally correlated and modulate both virulence and antifungal response
Source: PLoS Biol. 2021 Jun 1;19(6):e3001247. doi: 10.1371/journal.pbio.3001247 (PMC8168846; doi:10.1371/journal.pbio.3001247)
Supplement: Attachment — Submitted filename: Responses to reviewers.docx [file pbio.3001247.s018.docx]

No responses needed, all reviewers are satisfied.
